# CTA-determined tricuspid annular dilatation is associated with persistence of tricuspid regurgitation after transcatheter aortic valve replacement

**DOI:** 10.1007/s00392-023-02152-0

**Published:** 2023-01-13

**Authors:** Kornelia Löw, Julius Steffen, Hans Theiss, Martin Orban, Konstantinos D. Rizas, Magda Haum, Philipp M. Doldi, Lukas Stolz, Jonas Gmeiner, Christian Hagl, Steffen Massberg, Jörg Hausleiter, Daniel Braun, Simon Deseive

**Affiliations:** 1 Medizinische Klinik und Poliklinik I, LMU-Klinikum, Marchioninistr. 15, 81377 Munich, Germany; 2 Center for Cardiovascular Diseases (DZHK), Munich Heart Alliance, Partner Site German Munich, Munich, Germany; 3 Herzchirurgische Klinik und Poliklinik, Klinikum der Universität München, Munich, Germany

**Keywords:** Tricuspid annular dilatation, Tricuspid regurgitation, Transcatheter aortic valve replacement

## Abstract

**Aim:**

The aim of this study was to analyse the predictive value of CTA-determined tricuspid annular dilatation (TAD) on the persistence of tricuspid regurgitation (TR) in patients undergoing transcatheter aortic valve replacement (TAVR) for severe aortic stenosis (AS) and concomitant at least moderate TR.

**Methods and results:**

288 consecutive patients treated with TAVR due to severe AS and concomitant at least moderate TR at baseline were included in the analysis. As cutoff for TAD, the median value of the CTA-determined, to the body surface area-normalized tricuspid annulus diameter (25.2 mm/m^2^) was used. TAD had no impact on procedural characteristics or outcomes, including procedural death and technical or device failure according to the Valve Academic Research Consortium 3 criteria. However, the primary outcome of the study—TR persistence after TAVR was significantly more frequent in patients with compared to patients without TAD (odds ratio 2.60, 95% confidence interval 1.33–5.16, *p* < 0.01). Multivariable logistic regression analysis, adjusting for clinical and echocardiographic baseline characteristics, which are known to influence aetiology or severity of TR, confirmed TAD as an independent predictor of TR persistence after TAVR (adjusted odds ratio 2.30, 95% confidence interval 1.20–4.46, *p* = 0.01). Moreover, 2 year all-cause mortality was significantly higher in patients with persistence or without change of TR compared to patients with TR improvement (log-rank *p* < 0.01).

**Conclusion:**

In patients undergoing TAVR for severe AS and concomitant at least moderate TR at baseline, TAD is a predictor of TR persistence, which is associated with increased 2-year all-cause mortality.

**Graphical abstract:**

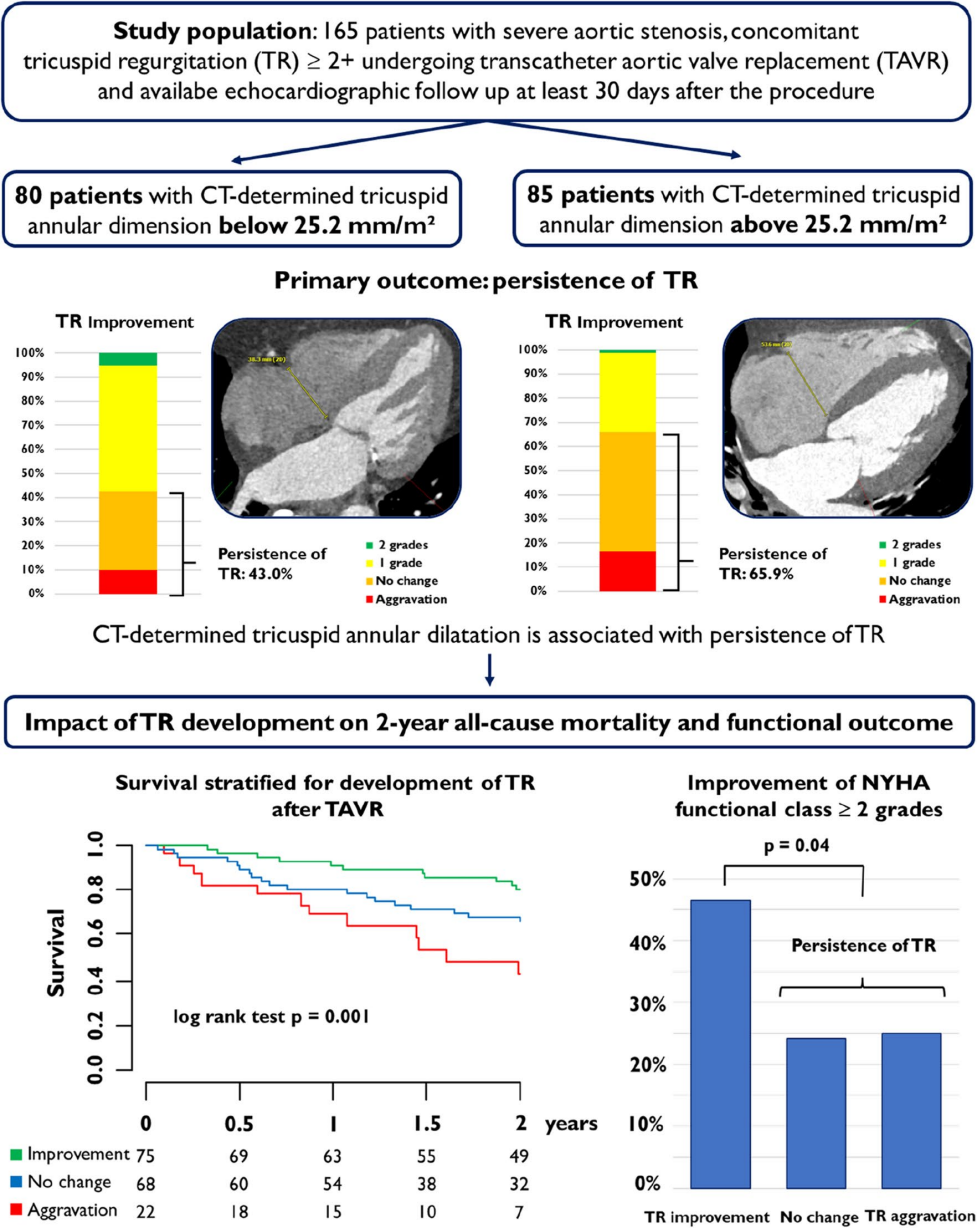

**Supplementary Information:**

The online version contains supplementary material available at 10.1007/s00392-023-02152-0.

## Introduction

Moderate or severe tricuspid regurgitation (TR) is observed in more than 25% of patients with severe aortic stenosis (AS). Most frequently, TR is of secondary aetiology and therefore often caused by left-sided heart disease [1, 2]. In patients with severe AS and high surgical risk due to age or comorbidities, transcatheter aortic valve replacement (TAVR) is the preferred treatment option, and concomitant TR is common in these patients [3, 4]. While guidelines recommend concomitant tricuspid valve surgery in patients undergoing left-sided heart surgery and at least moderate TR in the context of tricuspid annular dilatation, the best approach for treatment of TR in TAVR patients remains unknown [5]. Besides, an improvement of TR can be achieved after TAVR due to reduction of pressure overload in more than 50% of cases [6]. However, the persistence of TR after the procedure is associated with increased all-cause mortality [6, 7]. Therefore, it is of importance to identify determinants of TR persistence in patients undergoing TAVR.

Recently, computed tomography angiography (CTA)-determined tricuspid annular dilatation (TAD) proved to be an independent predictor of 2-year all-cause mortality in patients with severe AS undergoing TAVR [8]. The objective of this study was to investigate if CTA-determined TAD among TAVR patients who had at least moderate TR at baseline is associated with the persistence of TR after the procedure. Moreover, the predictive value of TR persistence on the composite of 2-year all-cause mortality in these patients was analysed.

## Methods

### Study design and population

Consecutive patients with severe AS and at least moderate concomitant TR at baseline who underwent TAVR at Munich University Hospital from April 2013 to December 2019 were included in this study. Patients with a history of previous tricuspid valve intervention or surgery and patients treated with TAVR for severe aortic regurgitation were excluded. Moreover, external preprocedural CTA was a criterion for exclusion to ensure a standardized imaging protocol.

Before TAVR, all patients were assessed by the local heart team, consisting of cardiac surgeons and interventional cardiologists. Data were collected in the context of the EVERY-Valve registry, which was approved by the local ethics committee of the University of Munich (project number 19-840).

### Tricuspid annular dilatation

Preprocedural multidetector computed tomography angiography was performed as part of the standard of care in all patients. To identify patients with TAD, the maximum septolateral diameter of the tricuspid annulus was measured and normalized to the body surface area (BSA) as described previously [8]. As cutoff for TAD, the median value of the CTA-determined BSA normalized tricuspid annulus diameter of all included patients was used.

### Echocardiographic analysis

Transthoracic echocardiography was performed before and after TAVR in accordance with the European and American guidelines [9, 10]. Moreover, a central in-house core laboratory analysis was carried out to assess echocardiographic parameters. Severity of AS was determined using the continuity equation method. To quantify TR, an integrated approach taking into account visual appearance, biplane vena contracta width, effective regurgitant orifice area (EROA) and regurgitant volume was used whenever possible. For TR grading, a five-grade scheme (mild, moderate, severe, massive, torrential) as proposed by Hahn et al. was applied [11]. TR aggravation was defined as an increase in TR severity and TR persistence as lack of TR improvement of at least one grade in the follow-up compared to the preprocedural echocardiography. Right ventricular function was assessed using tricuspid annular plane systolic excursion (TAPSE) and fractional area change.

### TAVR procedure

For all TAVR procedures, local anaesthetics were administered and a femoral access for TAVR implantation was used. Type and size of the prosthesis were selected considering patients’ characteristics and measurements of the aortic valve in preprocedural CTA by the interventional cardiologist. Pre- and/or post-dilatation was performed according to the operator’s discretion [8].

### Study endpoints

The primary endpoint of this study was persistence of TR after TAVR and was analysed in patients with available echocardiographic follow-up ≥ 30 days after the procedure. As secondary endpoints, 2-year all-cause mortality, tricuspid valve intervention, changes of echocardiographic parameters as well as functional status after TAVR using the New York Heart Association (NYHA) functional class were recorded.

Moreover, procedural outcomes of all patients with at least moderate TR, such as the composite endpoints technical failure (procedural death, cardiac structural complications, conversion to open surgery, prosthesis dislocation, 2nd valve prosthesis, immediate vascular surgery/intervention) and device failure at 30 days (technical failure, 30-day mortality, elevated mean pressure gradient, paravalvular regurgitation, vascular surgery/intervention) as well as early pacemaker implantation, stroke, bleeding and acute kidney injury according to the Valve Academic Research Consortium 3 were recorded [12].

### Statistical analysis

Continuous data are presented as median with interquartile range and categorical variables are expressed as frequencies and percentages. Differences between groups were tested for significance using the Fisher exact test, the Wilcoxon rank sum test or the Wilcoxon signed rank test as appropriate. 2-year all-cause mortality was evaluated using the Kaplan–Meier method and log-rank test. A two-sided *p* value < 0.05 was considered to indicate statistical significance. All statistical analyses were conducted using R version 4.0.2 (The R Foundation for Statistical Computing, Vienna, Austria).

## Results

### Study population

306 patients with severe AS and at least moderate concomitant TR, underwent TAVR procedure at Munich University Hospital between April 2013 and December 2019. 17 patients were excluded due to externally acquired preprocedural CTA, and one patient due to history of tricuspid valve surgery. The median value of the CTA-determined BSA normalized tricuspid annulus diameter of the remaining 288 patients was 25.2 mm/m^2^ and was used as cutoff to stratify patients into patients with (TAD +) and without TAD (TAD-).

### Procedural characteristics and outcomes

Regarding procedural characteristics, no differences could be observed between patients with and without TAD. Moreover, TAD had no impact on procedural outcomes, including procedural death (0.0% TAD − vs. 1.4% TAD + , *p* = 0.50), technical failure (6.9% TAD − vs. 5.6% TAD + , *p* = 0.81), device failure at 30 days (17.4% TAD − vs. 14.6% TAD + , *p* = 0.63), stroke (3.5% TAD − vs. 1.4% TAD + , *p* = 0.45) and bleeding BARC type 3 or 4 (15.3% TAD − vs. 11.1% TAD + , *p* = 0.38). Procedural characteristics and outcomes stratified by TAD are depicted in Table 1. In addition, all-cause mortality at 30 days was similar in both groups (*p* = 1.00),

**Table 1:** Procedural characteristics and outcomes

	All (*n* = 288)	TAD − (*n* = 144)	TAD + (*n* = 144)	*p*-value
Procedural characteristics				
Prosthesis type				p = 0.57
Sapien	209 (72.6)	104 (72.2)	105 (72.9)	
CoreValve	35 (12.2)	16 (11.1)	19 (13.2)	
Accurate Neo	14 (4.9)	10 (6.9)	4 (2.8)	
Lotus	21 (7.3)	10 (6.9)	11 (7.6)	
Other	9 (3.1)	4 (2.8)	5 (3.5)	
Prosthesis size				*p* = 0.53
< 25 mm	99 (34.4)	54 (37.5)	45 (31.3)	
25–28 mm	110 (38.2)	54 (37.5)	56 (38.9)	
> 28 mm	78 (27.1)	36 (25.0)	42 (29.2)	
Pre-dilatation performed	201 (69.8)	104 (72.2)	97 (67.4)	*p* = 0.37
Post-dilatation performed	16 (5.6)	8 (5.6)	8 (5.6)	*p* = 0.79
Procedural outcomes				
Technical failure	18 (6.3)	10 (6.9)	8 (5.6)	*p* = 0.81
Procedural death	2 (0.69)	0 (0.00)	1 (1.4)	*p* = 0.50
Cardiac structural complication	5 (1.7)	3 (2.1)	2 (1.4)	*p* = 1.00
Conversion to open surgery	2 (0.7)	0 (0.0)	2 (1.4)	*p* = 0.50
Prosthesis dislocation	4 (1.4)	3 (2.1)	1 (0.7)	*p* = 0.62
2nd valve prosthesis	0 (0.0)	0 (0.0)	0 (0.0)	*p* = 1.00
Immediate vascular surgery/intervention	10 (3.5)	5 (3.5)	5 (3.5)	*p* = 1.00
Device failure at 30 days	46 (16.0)	25 (17.4)	21 (14.6)	*p* = 0.63
30 day mortality	22 (7.6)	11 (7.6)	11 (7.6)	*p* = 1.00
Aortic regurgitation > 1 +	10 (3.5)	6 (4.2)	4 (2.8)	*p* = 0.75
Elevated PG mean > 20 mmHg	4 (1.4)	3 (2.1)	1 (0.7)	*p* = 0.62
Vascular intervention/surgery	11 (3.8)	5 (3.5)	6 (4.2)	*p* = 1.00
Early pacemaker implantation	48 (16.7)	30 (20.8)	18 (12.5)	*p* = 0.08
Stroke	7 (2.4)	5 (3.5)	2 (1.4)	*p* = 0.45
Bleeding BARC type 3 or 4	38 (13.2)	22 (15.3)	16 (11.1)	*p* = 0.38
Acute kidney injury stage 3 or 4	10 (3.5)	8 (5.6)	2 (1.4)	*p* = 0.10

*PG* pressure gradient, *BARC* bleeding academic research consortium

### Clinical and echocardiographic baseline characteristics

Out of 266 patients, who had survived at least 30 days after TAVR, echocardiographic follow-up ≥ 30 days after the procedure was available for 165 patients (62.0%) (median echocardiography follow-up time 101 days [interquartile range 52–342 days]). Comparing baseline characteristics between patients with and without available echocardiographic follow-up, patients without follow-up were older (82.2 years [IQR 77.5–86.0] patients with follow-up vs. 84.2 years [IQR 80.2–87.7] patients without follow-up, *p* < 0.01), but had a similar prevalence of comorbidities, including renal impairment, atrial fibrillation or coronary artery disease. Moreover, there was no difference regarding TR severity at baseline (TR grade ≥ 3: 30.3% in patients with vs. 29.3% in patients without follow-up, *p* = 0.90). Clinical and echocardiographic baseline characteristics comparing patients with and without follow-up are shown in Supplemental Table 1.

In patients with available echocardiographic follow-up, the BSA normalized tricuspid annulus diameter was above the threshold of 25.2 mm/m^2^ in 85 patients (TAD + group). Concerning clinical baseline characteristics, patients in the TAD + group were older (81.2 years [IQR 76.2–84.5] TAD − vs. 83.2 years [IQR 79.6–86.2] TAD + , *p* = 0.03) and suffered more often from atrial fibrillation (45.0% TAD − vs. 62.4% TAD + , *p* = 0.03) and renal impairment (47.5% TAD − vs. 70.6% TAD + , *p* < 0.01). Clinical baseline characteristics are presented in Table 2. Regarding echocardiographic parameters, severe AS with a median aortic valve orifice area of 0.7 cm^2^ was present in both groups. Dimensions of the right ventricle (RV) and right atrium (RA) were larger, and baseline TR was more pronounced in the TAD + group (TR grade ≥ 3: 20.0% TAD − vs. 40.0% TAD + , *p* = 0.05). Echocardiographic baseline characteristics are summarized in Table 3.

**Table 2:** Clinical baseline characteristics

	All (*n* = 165)	TAD − (*n* = 80)	TAD + (*n* = 85)	*p*-value
Clinical characteristics				
Male gender	76 (46.1)	36 (45.0)	40 (47.1)	*p* = 0.88
Age (years)	82.2 (77.5; 86.0)	81.2 (76.2; 84.5)	83.2 (79.6; 86.2)	*p* = 0.03
BMI (kg/m^2^)	24.7 (22.5; 27.7)	26.6 (24.2; 28.8)	22.9 (21.5; 24.9)	*p* < 0.01
STS score	4.8 (3.1; 7.8)	4.0 (3.0; 7.3)	5.0 (3.7; 8.5)	*p* = 0.08
NYHA functional class ≥ III	153 (93.3)	76 (95.0)	77 (91.7)	*p* = 0.54
Coronary artery disease	91 (57.6)	46 (59.7)	45 (55.6)	p = 0.63
Prior myocardial infarction	19 (11.7)	9 (11.3)	10 (12.0)	*p* = 1.00
Prior PCI	42 (25.6)	19 (23.8)	23 (27.4)	*p* = 0.72
Prior CABG	17 (10.4)	10 (12.5)	7 (8.3)	*p* = 0.45
Pacemaker or ICD	28 (17.0)	17 (21.3)	11 (12.9)	*p* = 0.21
Atrial fibrillation	89 (53.4)	36 (45.0)	53 (62.4)	*p* = 0.03
Renal impairment	98 (59.4)	38 (47.5)	60 (70.6)	*p* < 0.01
Diabetes	54 (32.7)	26 (32.5)	28 (32.9)	*p* = 1.00
Hypertension	144 (87.3)	73 (91.3)	71 (83.5)	*p* = 0.16
Smoking	30 (18.9)	12 (15.6)	18 (22.0)	*p* = 0.32
Hypercholesteremia	71 (44.4)	33 (42.9)	38 (45.8)	*p* = 0.75
NT-proBNP (pg/ml)	4040 (2179; 9736)	2911 (1518; 6265)	4324 (3234; 10034)	*p* = 0.09

*BMI* body mass index, *STS score* society of thoracic surgeons score, *NYHA* New York Heart Association, *PCI* percutaneous coronary intervention, *CABG* coronary artery bypass graft, *ICD* implantable cardioverter defibrillator, *NT-proBNP* N-terminal pro b-type natriuretic peptide

**Table 3:** Echocardiographic baseline characteristics

	All (*n* = 165)	TAD − (*n* = 80)	TAD + (*n* = 85)	*p*-value
Echocardiographic parameters				
LVEF (%)	53.2 (41.2; 58.6)	53.1 (40.7; 57.1)	53.6 (41.6; 59.5)	*p* = 0.45
PG max aortic valve (mmHg)	49.8 (38.0; 64.1)	48.4 (36.3; 62.1)	49.9 (38.1; 68.10)	*p* = 0.96
PG mean aortic valve (mmHg)	29.3 (22.0; 40.2)	28.6 (21.0; 39.5)	30.3 (23.0; 40.3)	*p* = 0.92
V max aortic valve (cm/s)	348.6 (300.9; 396.8)	341.0 (300.1; 391.5)	352.9 (308.5; 404.1)	*p* = 0.81
Stroke volume index (ml/m^2^)	29.9 (24.4;36.9)	30.2 (24.7; 35.3)	29.6 (23.9;38.2)	*p* = 0.79
Aortic valve orifice area (cm^2^)	0.7 (0.6; 0.9)	0.7 (0.6; 0.9)	0.7 (0.6; 0.8)	*p* = 0.26
Aortic regurgitation, *n* (%)				*p* = 0.33
Grade 0	24 (14.5)	15 (18.8)	9 (10.6)	
Grade 1	107 (64.8)	49 (61.3)	58 (68.2)	
Grade 2	34 (20.6)	16 (20.0)	18 (21.2)	
RV area change (%)	35.3 (30.0; 40.8)	36.3 (29.0; 40.7)	35.2 (30.0; 39.9)	*p* = 0.86
RV diameter at mid/BSA (mm/m^2^)	21.1 (18.7; 23.0)	19.9 (17.7; 22.4)	21.6 (19.9; 24.3)	*p* < 0.01
RV diameter at base/BSA (mm/m^2^)	27.7 (25.5; 31.3)	26.1 (24.4; 28.5)	29.6 (27.1; 32.6)	*p* < 0.01
Tricuspid annulus diameter/BSA (mm/m^2^)	22.0 (19.6; 24.6)	20.5 (18.5; 22.5)	23.7 (21.7; 25.6)	*p* < 0.01
Right atrium/BSA (cm^2^/m^2^)	15.1 (12.6; 19.0)	13.2 (11.1; 15.5)	17.2 (14.3; 20.4)	*p* < 0.01
TAPSE (mm)	17.0 (13.3; 19.8)	17.0 (14.0; 19.0)	17.0 (13.0; 20.0)	*p* = 0.69
TR vena contracta (mm)	6.2 (4.9; 8.3)	5.8 (4.7; 6.7)	6.5 (5.2; 10.0)	*p* = 0.01
TR EROA (mm^2^)	28.0 (21.0; 43.3)	26.0 (20.0; 32.0)	32.0 (21.5; 43.0)	*p* = 0.08
TR regurgitant volume (ml)	27.0 (21.0; 37.0)	25.0 (22.0;31.0)	29.0 (21.0; 43.0)	*p* = 0.15
dPmean TV inflow (mmHg)	1.0 (0.8; 1.4)	1.0 (0.8; 1.6)	1.0 (0.8; 1.2)	*p* = 0.17
RV/RA gradient (mmHg)	39.5 (28.9; 49.4)	43.4 (30.0; 49.9)	36.6 (28.0; 48.3)	*p* = 0.08
TR severity				*p* < 0.01
2	115 (69.7)	64 (80.0)	51 (60.0)	
3	39 (23.6)	15 (18.8)	24 (28.2)	
4	9 (5.5)	1 (1.3)	8 (9.4)	
5	2 (1.2)	0 (0.0)	2 (2.4)	
MR severity ≥ 2	88 (53.3)	40 (50.0)	48 (56.5)	*p* = 0.44
Vena cava inferior (mm)	22.0 (17.0; 25.0)	21.0 (17.0; 24.0)	22.0 (18.8; 25.0)	*p* = 0.12
Respiratory variance VCI	25 (33.8)	16 (51.6)	9 (20.9)	*p* = 0.01

*LVEF* left ventricular ejection fraction, *PG* pressure gradient, *V max* maximum velocity, *TV* tricuspid valve, *VCI* vena cava inferior

### Impact of tricuspid annular dilatation on tricuspid regurgitation

**Fig. 1:**
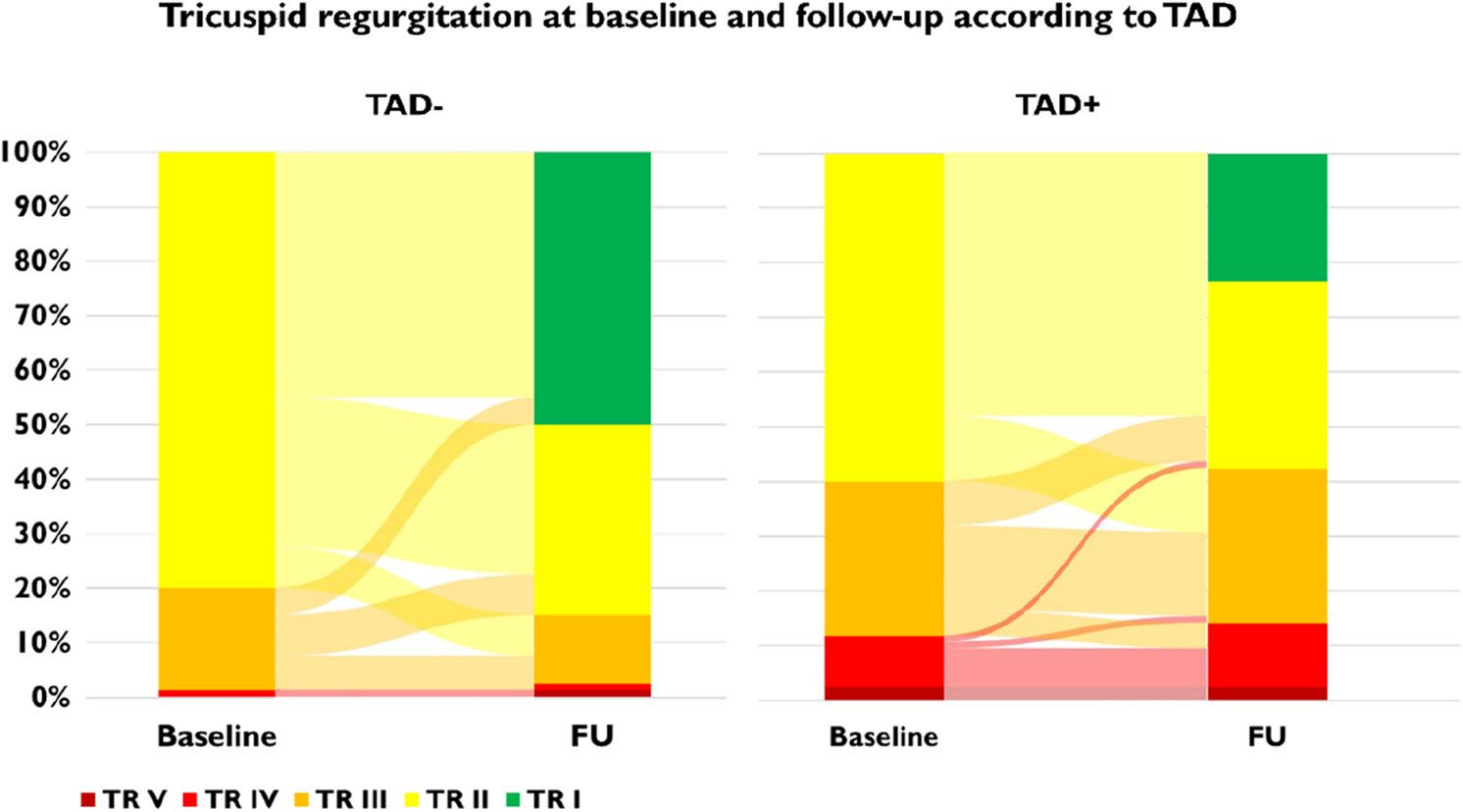
TR at baseline and follow-up according to TAD

**Fig. 2:**
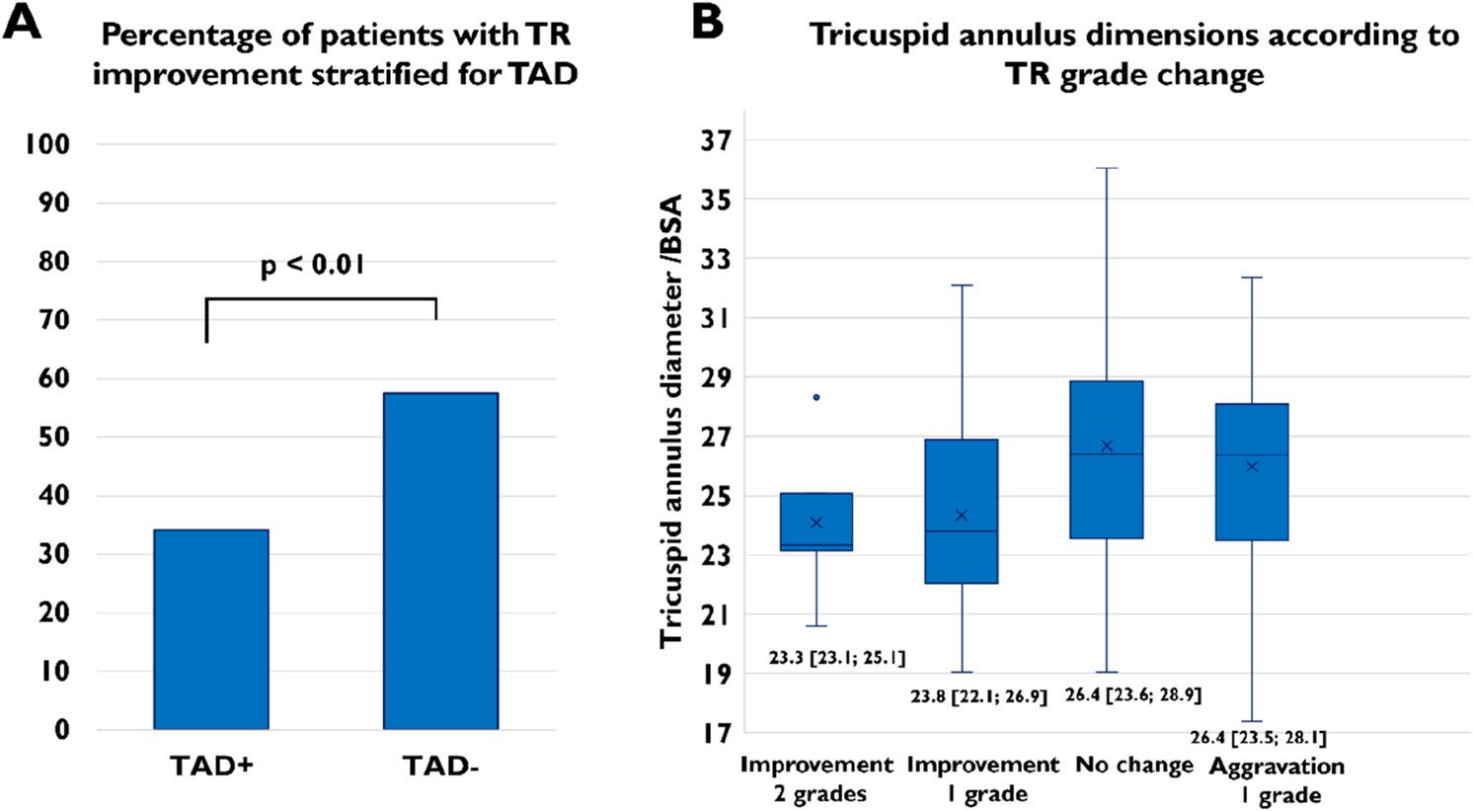
**A** Percentage of patients with TR improvement of at least one grade following TAVR stratified for TAD **B** Tricuspid annulus dimensions according to TR grade change

Improvement of TR after TAVR was observed in both groups, with a higher number of patients with TR grade 1 at follow-up in the TAD − group (TR grade 1 at follow-up: 50.0% TAD − vs. 23.5% TAD + , *p* < 0.01) (Fig. 1). Consistently, improvement of TR of at least one grade was significantly more frequent in patients in the TAD− group (57.0% TAD − vs. 34.1% TAD + , corresponding odds ratio for persistence of TR: 2.60, 95% confidence interval 1.33–5.16, *p* < 0.01) (Fig. 2A). The median value of the CTA-determined BSA normalized tricuspid annulus diameter was higher in patients with an aggravation or persistence of TR compared to patients with an improvement of TR of one or two grades (26.4 mm/m^2^ [IQR 23.6–28.7] vs. 23.7 mm/m^2^ [IQR 22.1–26.9], *p* < 0.001) (Fig. 2B). Moreover, we performed a multivariable logistic regression analysis with adjustment for clinical and echocardiographic parameters, that are known to influence aetiology or severity of TR, including atrial fibrillation, renal impairment, right ventricular lead, mitral regurgitation, baseline TR severity, RV/RA gradient and right ventricular function using TAPSE. Thus, the predictive value of TAD for TR persistence after TAVR (adjusted odds ratio 2.30, 95% confidence interval 1.20–4.46, *p* = 0.01) could be confirmed. Consistently, tricuspid valve intervention after TAVR was conducted in 11 patients, all of them in the TAD + group (12.9%).

Concerning echocardiographic parameters, we observed a similar decline of the aortic pressure gradient after TAVR in both groups. Consistent with TR improvement, the reduction of vena contracta width was significantly higher in patients without TAD compared to patients with TAD (− 2.0 mm [IQR − 3.9 to − 0.3] TAD − vs. 0.0 mm [IQR − 2.2–2.0] TAD + , *p* < 0.01). Moreover, we noted an increase in left ventricular ejection fraction at follow-up compared to baseline in patients in the TAD − group. Echocardiographic parameters at baseline and follow-up as well as delta values are presented in Table 4A–C.

**Table 4:** A–C Echocardiographic parameters at baseline and follow-up, (A) TAD −, (B) TAD + , (C) delta values

A TAD-	Baseline	FU	*p*-value
LVEF (%)	50.7 (35.5; 56.2)	54.5 (41.1; 58.7)	*p* = 0.03
PG max aortic valve (mmHg)	45.5 (31.4; 60.9)	13.9 (10.9; 19.2)	*p* < 0.01
PG mean aortic valve (mmHg)	27.0 (18.6; 39.2)	7.6 (6.0; 10.7)	*p* < 0.01
RV area change (%)	35.0 (28.9; 40.2)	37.8 (28.3; 44.5)	*p* = 0.20
RV diameter at mid/BSA (mm/m^2^)	19.7 (17.7; 22.4)	19.3 (16.2; 21.0)	*p* = 0.12
RV diameter at base/BSA (mm/m^2^)	26.0 (24.5; 28.1)	25.8 (24.3; 28.6)	*p* = 0.88
Tricuspid annulus diameter/BSA (mm/m^2^)	21.2 (18.6; 22.5)	19.9 (17.9; 21.7)	*p* = 0.25
Right atrium/BSA (cm^2^/m^2^)	13.1 (11.1; 15.7)	13.2 (11.4; 15.6)	*p* = 0.97
TAPSE (mm)	15.0 (13.0; 17.5)	16.0 (13.0; 20.0)	*p* = 0.19
TR vena contracta (mm)	6.1 (4.7; 8.1)	3.8 (2.5; 6.1)	*p* < 0.01
TR EROA (mm^2^)	31.0 (26.0; 33.0)	23.0 (20.0; 32.0)	*p* = 0.19
TR regurgitant volume (ml)	27.0 (25.0; 27.0)	21.5 (17.0; 26.3)	*p* = 0.20
dPmean TV inflow (mmHg)	1.2 (0.9; 1.8)	1.1 (0.9; 1.3)	*p* = 0.25
RV/RA gradient (mmHg)	40.6 (29.4; 47.7)	36.5 (28.3; 50.8)	*p* = 0.76
Vena cava inferior (mm)	22.0 (17.0; 24.0)	17.0 (16.0; 24.0)	*p* = 0.11

B TAD +	Baseline	FU	*p*-value
LVEF (%)	51.9 (37.1; 58.4)	51.5 (43.2; 57.9)	*p* = 0.40
PG max aortic valve (mmHg)	47.7 (33.8; 58.1)	14.8 (10.1; 18.2)	*p* < 0.01
PG mean aortic valve (mmHg)	29.8 (20.9; 36.7)	7.9 (5.4; 9.9)	*p* < 0.01
RV area change (%)	35.2 (30.4; 37.5)	37.0 (31.0; 43.0)	*p* = 0.17
RV diameter at mid/BSA (mm/m^2^)	22.0 (20.1; 24.8)	20.7 (18.8; 23.7)	*p* = 0.32
RV diameter at base/BSA (mm/m^2^)	30.7 )27.2; 32.8]	28.8 )26.4; 32.7]	*p* = 0.15
Tricuspid annulus diameter/BSA (mm/m^2^)	24.3 (23.0; 26.2)	22.5 (20.9; 25.1)	*p* = 0.01
Right atrium/BSA (cm^2^/m^2^)	17.2 )14.5; 20.3]	17.1 )13.8; 20.3]	*p* = 0.11
TAPSE (mm)	15.0 (12.3; 19.8)	16.0 (13.0; 19.0)	*p* = 0.51
TR vena contracta (mm)	6.3 (5.0; 9.2)	6.3 )4.4; 10.0]	*p* = 0.77
TR EROA (mm^2^)	31.0 (23.5; 50.0)	30.0 (21.0; 49.3)	*p* = 0.56
TR regurgitant volume (ml)	29.0 (21.0;45.0)	31.5 (21.3; 40.8)	*p* = 0.83
dPmean TV inflow (mmHg)	1.0 (0.7; 1.2)	1.0 (0.8; 1.4)	*p* = 0.41
RV/RA gradient (mmHg)	35.3 (25.5; 45.5)	32.7 (27.8; 41.6)	*p* = 0.42
Vena cava inferior (mm)	21.0 (17.0; 25.0)	21.0 (18.0; 26.0)	*p* = 0.93

C	TAD −	TAD +	*p* value
Δ LVEF (%)	3.6 (− 1.4; 5.7)	− 0.3 (− 4.8; 6.7)	*p* = 0.34
Δ PG max aortic valve (mmHg)	− 29.0 (− 42.7; − 20.8)	− 34.2 (− 44.8; − 16.9)	*p* = 0.93
Δ PG mean aortic valve (mmHg)	− 18.0 (− 31.2; − 10.4)	− 21.5 (− 29.6; − 11.4)	*p* = 0.59
Δ RV area change (%)	3.1 (− 3.2; 5.5)	3.2 (− 7.0; 7.6)	*p* = 0.74
Δ RV diameter at mid (mm/ m^2^)	− 0.6 (− 3.6; 1.5)	− 1.2 (− 3.4; 2.3)	*p* = 0.63
Δ RV diameter at base (mm/ m^2^)	0.0 (− 2.4; 3.2)	− 0.7 (− 3.5; 2.0)	*p* = 0.32
Δ Tricuspid annulus diameter/BSA (mm/m^2^)	− 0.3 (− 1.9; 1.0)	− 1.2 (− 3.3; 1.1)	*p* = 0.30
Δ Right atrium (cm^2^/m^2^)	0.5 (− 2.0; 1.4)	− 0.5 (− 3.2; 1.3)	*p* = 0.29
Δ TAPSE (mm)	1.0 (− 1.0; 3.0)	− 1.0 (− 3.0; 2.0)	*p* = 0.13
Δ TR vena contracta (mm)	− 2.0 (− 3.9; − 0.3)	0.0 (− 2.2; 2.0)	*p* < 0.01
Δ TR EROA (mm^2^)	− 7.0 [− 13.0; − 4.0]	− 2.5 (− 11.3; 6.8)	*p* = 0.28
Δ TR regurgitant volume (ml)	− 8.0 (− 9.8; 1.3)	− 2.0 (− 8.8; 8.8)	*p* = 0.70
Δ dPmean TV inflow (mmHg)	− 0.2 (− 0.5; 0.3)	0.0 (− 0.3; 0.3)	*p* = 0.12
Δ RV/RA gradient (mmHg)	0.0 (− 12.1; 8.5)	0.9 (− 9.8; 7.2])	*p* = 0.91
Δ Vena cava inferior (mm)	− 2.0 (− 6.0; 2.0)	0.0 (− 4.0; 3.0)	*p* = 0.31

*LVEF* left ventricular ejection fraction, *PG* pressure gradient, *TV* tricuspid valve

### Impact of TR development on survival and functional status

**Fig. 3:**
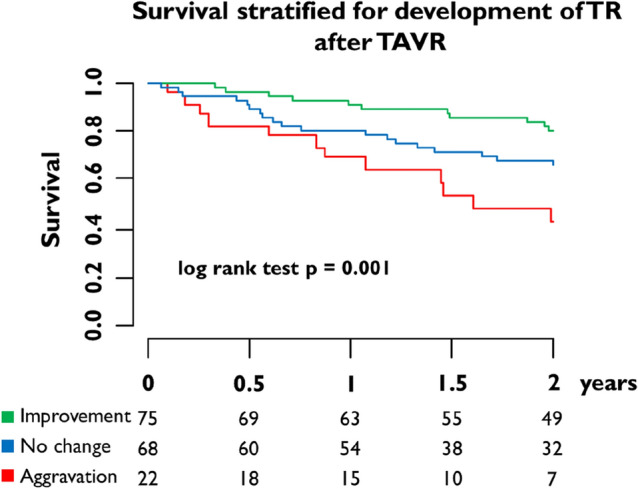
Survival stratified for development of TR after TAVR

**Fig. 4:**
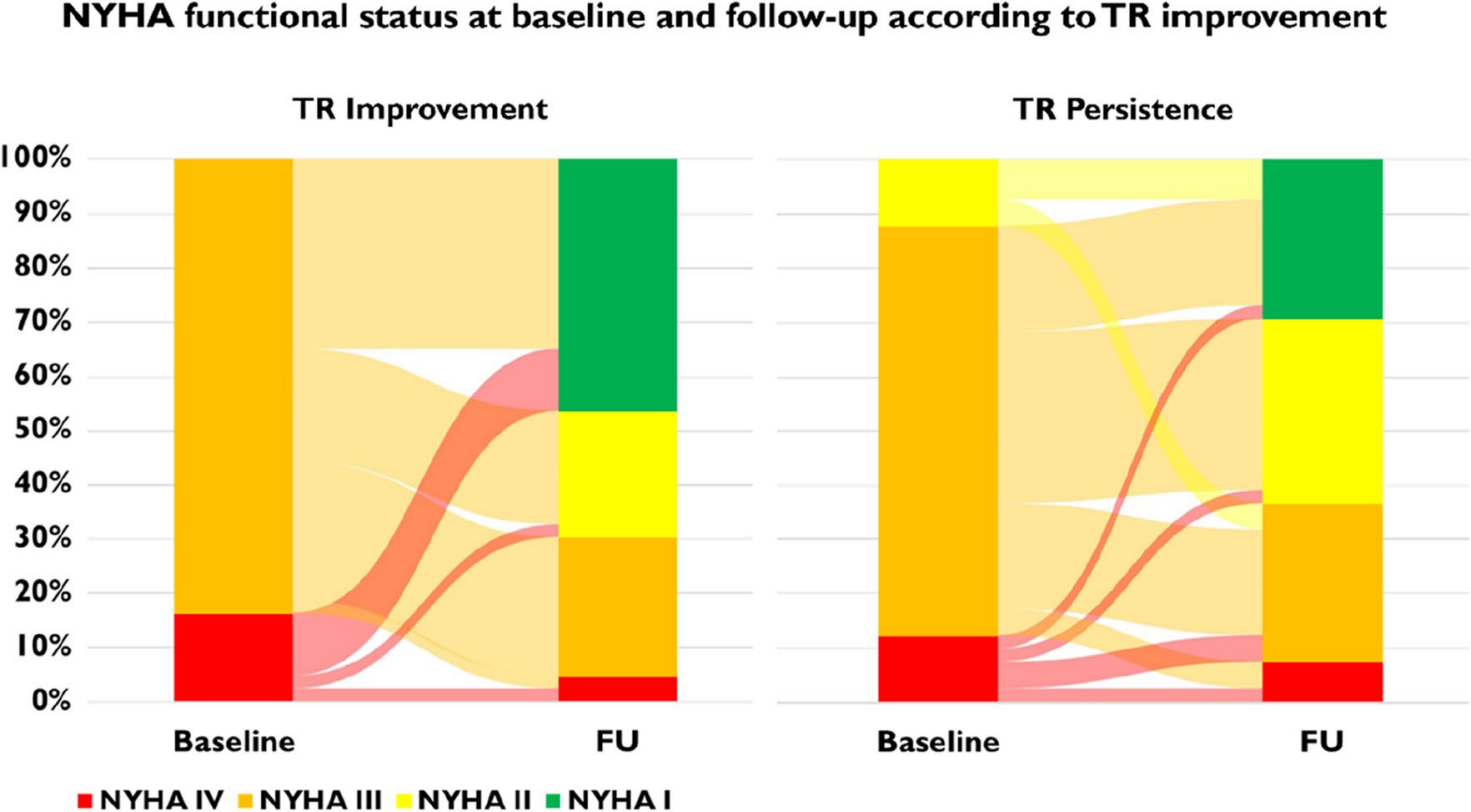
NYHA functional status at baseline and follow-up according to TR improvement

Two-year follow-up information was available in 81.8% of patients. All-cause mortality was significantly lower in patients with improvement of TR compared to patients without change or aggravation of TR (log-rank *p* < 0.01). The corresponding hazard ratio for 2-year all-cause mortality in patients with TR improvement vs. no change of TR and vs. aggravation was 0.47 (95% confidence interval 0.24 to 0.94) and 0.24 (95% confidence interval 0.11 to 0.54), respectively. Kaplan–Meier curves are shown in Fig. 3. Concerning functional status, improvement of NYHA functional class of at least two grades after the procedure was observed less often in patients with persistence of TR compared to patients with TR improvement (24.4 vs. 46.5%, *p* = 0.04) (Fig. 4).

## Discussion

Our analysis demonstrates that in patients undergoing TAVR for severe AS and at least moderate concomitant TR at baseline, CTA-derived TAD is associated with the persistence of TR after the procedure. Consistently, no patient without TAD required tricuspid valve treatment within the 2 year follow-up period. Besides, TR persistence is associated with increased 2 year all-cause mortality.

While former studies stated an association between significant TR at baseline and all-cause mortality in patients with AS undergoing TAVR, recent analyses demonstrated that TR persistence after the procedure is associated with increased mortality and might therefore be prognostically more relevant than TR severity at baseline [6, 7, 13, 14]. In this study, we could confirm that in TAVR patients with concomitant at least moderate TR at baseline, TR persistence is associated with increased all-cause mortality after 2 years. In addition, improvement of NYHA functional class of at least two grades was observed less often in patients with persistence of TR. A multivariable logistic regression analysis with adjustment for atrial fibrillation, renal impairment, right ventricular lead, mitral regurgitation, baseline TR severity, RV/RA gradient and right ventricular function confirmed that TAD is an independent predictor of TR persistence. Moreover, no difference regarding procedural characteristics and outcomes, including procedural mortality, technical or device failure was found between patients with and without TAD. Hence, an impact of procedural factors on the differences in outcomes seems unlikely.

As the preprocedural CTA is part of the standard of care to evaluate vascular access routes and to enable accurate prosthesis selection, the tricuspid annulus diameter can be obtained easily without further diagnostic effort. In addition, the measurement is less error-prone and with a lower degree of interobserver variability compared to echocardiographic assessment. In transthoracic echocardiography, an optimal acoustic window of the RV in the RV-focused apical four-chamber view is necessary to obtain the dimensions of the tricuspid valve. Therefore, and due to the complex oval and saddle-shaped anatomy of the valve, its maximal diameter is often underestimated in echocardiography. On the contrary, datasets of CT-scans can be angulated precisely in the tricuspid annulus for exact assessment of its dimensions as described previously [8]. Hence, CTA-determined BSA normalized tricuspid annulus diameter can serve as a reliable and easily accessible parameter to predict persistence of TR in patients with severe AS treated with TAVR.

Although concomitant tricuspid valve surgery is recommended in patients undergoing left-sided heart surgery and at least moderate TR in the context of tricuspid annular dilatation, optimal management of TR in TAVR patients remains unknown [5]. Considering the fact that moderate or severe TR can be observed in more than 25% of patients with severe AS and that the number of TAVR procedures will increase due to favorable outcomes in recent studies for asymptomatic or low-risk patients, this question might be even of higher relevance in the future [4, 15–17].

While sufficient literature regarding tricuspid valve intervention for persistent TR after TAVR is scarce, a recently published propensity-matched case–control study could demonstrate a benefit for patients without AS and at least moderate TR. Patients treated with transcatheter tricuspid valve intervention had significantly lower rates of mortality and rehospitalization compared to medically managed patients [18]. Besides, the less invasive nature of transcatheter valve repair and replacement procedures compared to open-heart surgery could facilitate a watch-and-wait strategy. Therefore, TAD could serve not only as a predictor of TR persistence after TAVR, but also as a tool to identify patients in need for intensified post-TAVR echocardiographic and clinical surveillance. In case of TR persistence and lack of symptomatic improvement, these patients might be candidates for transcatheter tricuspid valve interventions. Moreover, since tricuspid valve interventions especially edge-to-edge repair evolved in the last years and gained importance recently, the percentage of patients undergoing these procedures might even be higher in the future and TAD could serve as a tool to identify these patients.

### Study limitations

The retrospective nature and the incomplete echocardiographic follow-up are major limitations of this study, as it  poses a selection bias. Furthermore, we performed a central core laboratory analysis for the assessment of echocardiographic parameters, but analysis was limited by the available echocardiographic images and especially 3D volumetric data were not routinely recorded.

## Conclusions

In patients undergoing TAVR for severe AS with at least moderate concomitant TR, TAD identifies patients with persistence of TR after the procedure, which is associated with increased 2-year all-cause mortality.

## Supplementary Information

Below is the link to the electronic supplementary material.

Supplementary file1 (DOCX 17 KB)

## Abbreviations

AS: Aortic stenosis
BARC: Bleeding academic research consortium
BSA: Body surface area
CTA: Computed tomography angiography
EROA: Effective regurgitant orifice area
NYHA: New York Heart Association
RA: Right atrium
RV: Right ventricle
TAD: Tricuspid annular dilatation
TAPSE: Tricuspid annular plane systolic excursion
TAVR: Transcatheter aortic valve replacement
TR: Tricuspid regurgitation
