# Supplementary material for: CTA-determined tricuspid annular dilatation is associated with persistence of tricuspid regurgitation after transcatheter aortic valve replacement
Source: Clin Res Cardiol. 2023 Jan 13;112(5):645–55. doi: 10.1007/s00392-023-02152-0 (PMC10160207; doi:10.1007/s00392-023-02152-0)
Supplement: Supplementary file 1 — Supplementary file1 (DOCX 17 KB) [file 392_2023_2152_MOESM1_ESM.docx]

**Supplemental Table 1*:* Clinical and echocardiographic baseline characteristics comparing patients with and without follow up.**

|  | FU available  (n = 165) | FU not available  (n =123) | p-value |
| --- | --- | --- | --- |
| Male gender | 76 (46.1) | 51 (41.4) | p = 0.47 |
| Age (years) | 82.2 [77.5; 86.0] | 84.2 [80.2; 87.7] | p < 0.01 |
| BMI (kg/m²) | 24.7 [22.5; 27.7] | 24.6 [22.2; 27.7] | p = 0.97 |
| STS score | 4.8 [3.1; 7.8] | 5.3 [3.0; 9.0] | p = 0.44 |
| NYHA functional class ≥ III | 153 (93.3) | 102 (91.1) | p = 0.50 |
| Coronary artery disease | 91 (57.6) | 73 (64.6) | p = 0.26 |
| Prior myocardial infarction | 19 (11.7) | 20 (17.9) | p = 0.16 |
| Prior PCI | 42 (25.6) | 32 (28.8) | p = 0.58 |
| Prior CABG | 17 (10.4) | 11 (9.8) | p = 1.00 |
| Atrial fibrillation | 89 (53.4) | 69 (56.1) | p = 0.81 |
| Renal impairment | 98 (59.4) | 75 (61.0) | p = 0.90 |
| Diabetes | 54 (32.7) | 33 (29.2) | p = 0.60 |
| Hypertension | 144 (87.3) | 101 (89.4) | p = 0.71 |
| Smoking | 30 (18.9) | 16 (14.7) | p = 0.41 |
| Hypercholesteremia | 71 (44.4) | 41 (36.6) | p = 0.21 |
| NT-proBNP (pg/ml) | 4040 [2179; 9736] | 2771 [1641; 11193] | p = 0.50 |
| LVEF (%) | 53.2 [41.2; 58.6] | 55.0 [44.8; 55.0] | p = 0.64 |
| PG max Aortic valve (mmHg) | 49.8 [38.0; 64.1] | 52.0 [40.0; 65.0] | p = 0.37 |
| PG mean Aortic valve (mmHg) | 29.3 [22.0; 40.2] | 30.0 [23.3; 38.8] | p = 0.60 |
| RV area change (%) | 35.3 [30.0; 40.8] | 33.9 [27.0; 41.5] | p = 0.45 |
| TAPSE (mm) | 17.0 [13.3; 19.8] | 18.0 [14.0; 22.0] | p = 0.04 |
| TR vena contracta (mm) | 6.2 [4.9; 8.3] | 5.8 [4.7; 7.6] | p = 0.22 |
| TR EROA (mm²) | 28.0 [21.0; 43.3] | 23.5 [22.8; 48.8] | p = 0.25 |
| RV/RA gradient (mmHg) | 39.5 [ 28.9; 49.4] | 43.5 [35.3; 53.8] | p < 0.01 |
| TR severity ≥ 3 | 50 (30.3) | 36 (29.3) | p = 0.90 |
